# Physical Exercise and Spatial Training: A Longitudinal Study of Effects on Cognition, Growth Factors, and Hippocampal Plasticity

**DOI:** 10.1038/s41598-018-19993-9

**Published:** 2018-03-09

**Authors:** Luise Woost, Pierre-Louis Bazin, Marco Taubert, Robert Trampel, Christine L. Tardif, Alexander Garthe, Gerd Kempermann, Ulrich Renner, Günter Stalla, Derek V. M. Ott, Viola Rjosk, Hellmuth Obrig, Arno Villringer, Elisabeth Roggenhofer, Tilmann A. Klein

**Affiliations:** 1 Department of Neurology, Max Planck Institute for Human Cognitive and Brain Sciences, Leipzig, Germany; 2 Social Brain Laboratory, Netherlands Institute for Neuroscience, Amsterdam, Netherlands; 3 Spinoza Centre for Neuroimaging, Amsterdam, Netherlands; 4 Department of Neurophysics, Max Planck Institute for Human Cognitive and Brain Sciences, Leipzig, Germany; 5 Department of Sport Science, Otto von Guericke University, Magdeburg, Germany; 6 Center for Behavioral Brain Sciences, Magdeburg, Germany; 7 McConnell Brain Imaging Centre, Montreal Neurological Institute, Montreal, Quebec, Canada; 8 Departments of Neurology and Neurosurgery, and Biomedical Engineering, McGill University, Montreal, Quebec, Canada; 9 German Center for Neurodegenerative Diseases, Dresden, Germany; 10 Center for Regenerative Therapies, Technical University, Dresden, Germany; 11 Department of Clinical Research, Max Planck Institute for Psychiatry, Munich, Germany; 12 Epilepsy Center Berlin-Brandenburg, Berlin, Germany; 13 Clinic for Cognitive Neurology, University Hospital of Leipzig, Leipzig, Germany; 14 Center for Stroke Research Berlin, Charité - Universitätsmedizin Berlin, Berlin, Germany; 15 Neurology Division, Department of Clinical Neurosciences, Geneva University Hospitals, Geneva, Switzerland; 16 Institute of Psychology, Otto von Guericke University, Magdeburg, Germany

## Abstract

Physical exercise has been suggested to improve cognitive performance through various neurobiological mechanisms, mediated by growth factors such as BDNF, IGF-I, and VEGF. Moreover, animal research has demonstrated that combined physical and cognitive stimulation leads to increased adult neurogenesis as compared to either experimental condition alone. In the present study, we therefore investigated whether a sequential combination of physical and spatial training in young, healthy adults elicits an additive effect on training and transfer gains. To this end, we compared the effects of (i) eight 20-minute sessions of cycling, (ii) sixteen 30-minute sessions of spatial training, (iii) a combination of both, and included (iv) a passive control cohort. We assessed longitudinal changes in cognitive performance, growth factor levels, and T_1_ relaxation of hippocampal subfields (acquired with 7 T MRI). While substantial physical and spatial training gains were elicited in all trained groups, longitudinal transfer changes did not differ between these groups. Notably, we found no evidence for an additive effect of sequential physical and spatial training. These results challenge the extrapolation from the findings reported in animals to young, healthy adults.

## Introduction

Physical exercise has been associated with improved performance in various cognitive domains, including processing speed and attention^1^, intelligence^2^, spatial learning^3^, novel object recognition memory^4^, cognitive flexibility^5^, and also vocabulary learning^6^. Additionally, self-reported physical activity was found to have protective effects on gray matter volume in later life, thereby reducing the risk of cognitive impairment^7^. However, much of what is known about neurobiological fundamentals of exercise-induced effects on cognitive performance rests on animal research. In the adult mouse hippocampus, physical exercise increases neurogenesis^8^, synaptogenesis^9^, and long-term potentiation (LTP)^10^. Moreover, exercise-evoked increase in cerebral blood volume (CBV) in the human dentate gyrus (DG) has been suggested to be an *in-vivo* correlate of adult neurogenesis^11^. However, adult neurogenesis only partially explains exercise-induced effects on brain structure and function in humans. For example, changes in tissue density^12^ and myelination^13^ were discussed to act as additional candidate mechanisms that underlie exercise-related volume changes in the human hippocampus. Moreover, exercise-related changes in brain structure and function are mediated by various growth factors such as brain-derived neurotrophic factor (BDNF), insulin-like growth factor-I (IGF-I), and vascular endothelial growth factor (VEGF)^14^. It has been shown that BDNF promotes LTP^15^, myelination^16^, and neuronal differentiation^17^, while IGF-I stimulates BDNF expression^18^, neurogenesis^19^, and vessel remodeling^20^. VEGF has been observed to induce neurogenic effects^21^, angiogenesis, and LTP^22^.

Interestingly, animal studies have suggested that the combination of running and environmental enrichment leads to an additive effect on neurogenesis in the adult DG^23^. This has been ascribed to the interaction of pro-proliferative effects induced by running and survival-promoting effects caused by subsequent cognitive stimulation^23^. Adult neurogenesis in the hippocampus may hence be a component of the brain response to physical exercise with learning enhancing integration of new neurons in the hippocampal circuitry and survival of these neurons. Proceeding from such evidence, the present study aimed at investigating potentially additive effects of combined physical exercise and spatial training in young, healthy adults. To this end, 99 subjects were assigned to four subgroups completing (i) eight 20-minute sessions of cycling (group ‘ERGO’), (ii) sixteen 30-minute sessions of spatial training (group ‘MAZE’), (iii) a combination of both (group ‘COMBO’), or resting as (iv) passive controls (group ‘CTR’). To our knowledge, this is the first study to explore a strictly sequential rather than simultaneous or interleaved combination of different training regimes in humans. Since the physical exercise was finished prior to the onset of the spatial training (group COMBO), we addressed sustained rather than acute effects of enhanced physical activity on subsequent spatial training. Additionally, we looked into longitudinal transfer changes by repeated measurements at baseline (T0), after physical exercise (T1) and spatial training (T2), and after a non-intervention period (T3). At each time point, various plasticity-related transfer measures were acquired, including cognitive performance, serum levels of BDNF, IGF-I and VEGF, and longitudinal relaxation times T_1_ of 12 hippocampal subfields using 7 T Magnetic Resonance Imaging (MRI). Hippocampal subfields included left and right entorhinal cortex (ERC), subiculum (SUB), cornu ammonis (CA) subfield 1 (CA1), CA2, CA3, and DG/CA4. Longitudinal relaxation describes the regrowth of the longitudinal magnetization M_z_ after spin excitation and is characterized by the time constant T_1_. As longitudinal relaxation is affected by the presence of macro-molecules, in the healthy human brain T_1_ mainly reflects variations in myelin content (90% in white matter and 64% in gray matter, although this may vary between brain regions), but with a modest contribution from iron^24^. Furthermore, as the technique is quantitative, it is independent of the specific hardware (other than field strength), its values are reproducible and depend only on the underlying tissue sub-structure. Therefore, T_1_ mapping provides a less confounded MRI measure of brain plasticity compared to the more conventional T_1_ weighting^25^.

## Results

### Study Sample

**Figure 1:**
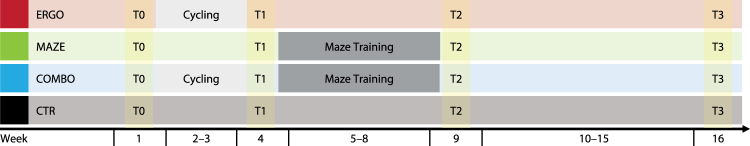
Overview of the Study Design and Time Points of Assessment (T0–T3). Participants attended one of four experimental conditions (ERGO, MAZE, COMBO, or CTR). Between time points T0 and T1, groups ERGO and COMBO completed eight 20-minute sessions of cycling while groups MAZE and CTR rested as passive controls. Between time points T1 and T2, groups MAZE and COMBO completed sixteen 30-minute sessions of maze training while groups ERGO and CTR rested as passive controls. No training took place between time points T2 and T3. At each assessment time point, participants took part in cognitive assessment, blood sampling, and 7 T MRI. Note that CTR only completed the cognitive assessment in order to control for test-retest effects induced by repeated testing. COMBO = group undergoing cycling exercise and maze training, CTR = passive controls, ERGO = group undergoing cycling exercise, MAZE = group undergoing maze training, MRI = Magnetic Resonance Imaging.

The final sample consisted of n = 99 young (60 females, 39 males) volunteers aged 20 to 34 years (M = 25.24, SD = 3.55). As revealed by analysis of variance (ANOVA) and chi-square test, respectively, groups did not significantly differ at baseline T0 regarding age, depressive symptoms as assessed with Beck Depression Inventory-II (BDI-II)^26^, sex, level of education, and smoking habits (p ≥ 0.153; see Table 1). Figure 1 provides a sketch of the study design and time points of assessment, details are provided in the Methods section.

**Table 1:** Sample Characteristics at Baseline (T0).

Measure	CTR (n = 26)	ERGO (n = 26)	MAZE (n = 23)	COMBO (n = 24)
Age (years), M (SD)	26.12 (3.42)	25.12 (3.91)	23.91 (3.52)	25.71 (3.09)
BDI-II, M (SD)	3.38 (3.26)	2.23 (2.54)	1.83 (1.67)	2.29 (2.63)
Women (%)	57.69	53.85	69.57	62.50
Abitur (%)	96.15	96.15	100.00	95.83
Smokers (%)	30.77	26.92	17.39	12.50

As revealed by analysis of variance (ANOVA) and chi-square test, respectively, groups did not differ regarding baseline characteristics. ‘Abitur’ denotes the German version of the university entrance qualification. BDI-II = Beck Depression Inventory-II, COMBO = group undergoing cycling exercise and maze training, CTR = passive controls, ERGO = group undergoing cycling exercise, MAZE = group undergoing maze training.

### Direct Effects of the Applied Training Regimes

#### Change in Physical Working Capacity (PWC) Induced by the Cycling Exercise

**Figure 2:**
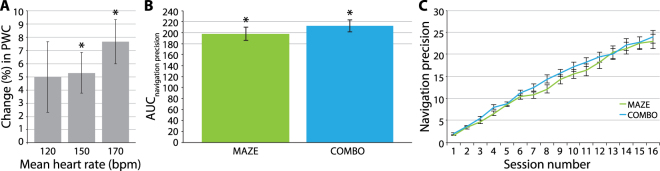
Direct Effects of the Applied Training Regimes. **(A)** Percent Change (Mean ± SEM) in PWC at Mean Heart Rates of 120, 150, and 170 bpm. PWC was defined by determining pedal resistance in watts (W) at predefined mean heart rates (120, 150, and 170 bpm), with *W · kg*_*baseline*_^−1^. Positive values indicate an increase in PWC. Values were collapsed over ERGO and COMBO (see Methods). For the mean heart rates of 150 and 170 bpm, percent change in PWC was substantially different from zero. For the mean heart rate of 120 bpm, percent change did not reach statistical significance. **(B)** Maze Training Gain (Mean ± SEM). Maze training gain was defined by estimating the AUC for navigation precision over session number, with *navigation precision* = *(path length*_*optimum*_ *· difficulty) · mean path length*_*subject*_^−1^. Navigation precision was determined for the most difficult, yet successfully completed level per session. Higher values indicate greater navigation precision. Both groups, MAZE and COMBO, showed substantial maze training gain. However, maze training gain did not differ between these groups. **(C)** Navigation Precision over Session Number (Mean ± SEM). Change in navigation precision over session number did not differ between groups MAZE and COMBO. AUC = area under the curve, bpm = beats per minute, COMBO = group undergoing cycling exercise and maze training, ERGO = group undergoing cycling exercise, MAZE = group undergoing maze training, PWC = physical working capacity, *p < 0.05 (A: after applying Bonferroni correction).

To determine the effectiveness of the cycling exercise, we tested the pre- to post-cycling change in PWC (i.e. cycling gain) by using one-sample t-tests. PWC was defined by assessing pedal resistance in watts (W) at predefined mean heart rates of 120 (PWC120), 150 (PWC150), and 170 bpm (PWC170). As groups ERGO and COMBO attended identical exercise sessions, we collapsed this analysis over both groups. Physical exercise via high-intensity training elicited substantial change in weight-adapted PWC. This applied to both PWC150 and PWC170 (t_45_ ≥ 3.441, p ≤ 0.001, one-sample t-test), whereas change in PWC120 did not reach statistical significance (t_43_ = 1.864, p = 0.069, one-sample t-test; see Fig. 2A). Results were obtained after exclusion of outliers (see Methods for details; see Supplementary Table S2 for outliers) and after correction for multiple comparisons using the Bonferroni method. In sum, a substantial gain in PWC was induced by the cycling exercise.

#### Change in Navigation Precision Induced by the Maze Training

To determine the effectiveness of the maze training, we analyzed maze training gain, measured as estimated area under the curve (AUC) for navigation precision over session number (see Methods), by using a one-sample t-test. According to our main hypothesis, which assumes differences in maze training gain between groups MAZE and COMBO, this analysis was conducted group-wise. MAZE and COMBO both revealed considerable maze training gain (t_21/22_ ≥ 16.432, p = 0.000). However, no significant group difference was found for this measure of training gain (t_43_ = −0.905, p = 0.371, independent-samples t-test; see Fig. 2B). In line with this, change in navigation precision over session number did not significantly vary between groups MAZE and COMBO (group × time: Greenhouse-Geisser adjusted F_4.17, 179.37_ = 0.669, p = 0.620, repeated measures ANOVA; see Fig. 2C). Hence, the overall direct gain induced by spatial training was not further augmented by prior physical exercise.

### Transfer Effects of the Applied Training Regimes

#### Effects on Longitudinal Transfer Changes

So far, it was demonstrated that both the cycling exercise and maze training per se were highly effective in inducing effects on directly related performance metrics. Next, we analyzed whether these direct effects transferred to related domains such as cognitive performance, growth factor levels, and hippocampal plasticity by checking for group differences in transfer change over time. Results were obtained by applying linear mixed modeling to each variable of interest, including 15 cognitive performance scores, serum BDNF, IGF-I and VEGF, and median T_1_ relaxation times of 12 hippocampal subfields (see Methods). The critical effect investigated with this analysis is revealed by a significant interaction of group by time.

**Figure 3:**
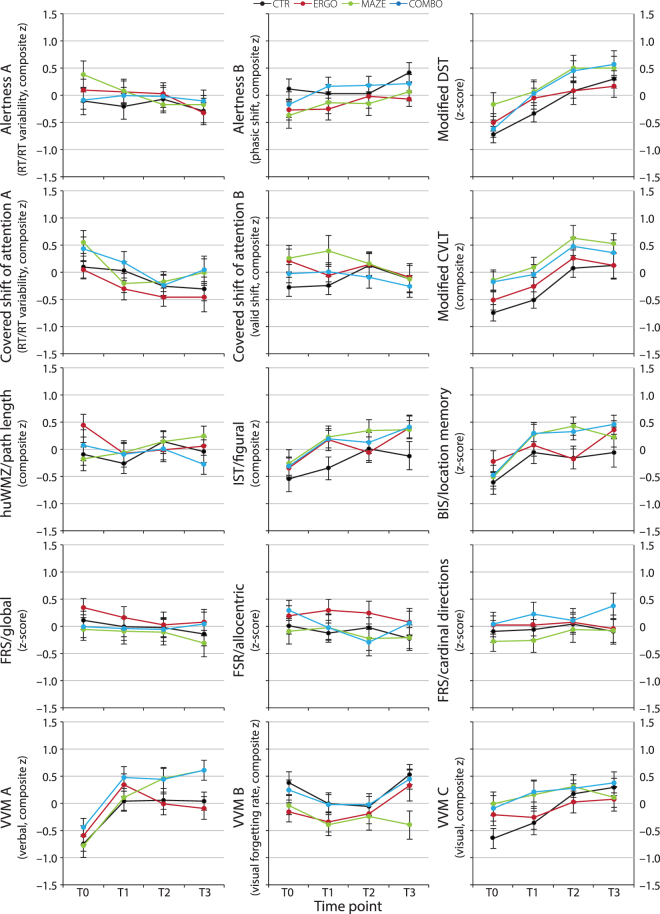
Group Means (± SEM) of Cognitive Performance Scores at Time Points T0–T3. FRS subscales, DST, and BIS represent z-scores, obtained across groups and time points after exclusion of outliers. Remaining values show composite z-scores, determined based on principal component analysis (PCA; see Methods and Supplementary Information). We did not observe group differences in linear change over time. Note that we applied modified versions of DST and CVLT (see Methods). Alertness = subtest ‘Alertness’ from ‘Tests of Attentional Performance’ (TAP), BIS = ‘Berlin Intelligence Structure Test’, COMBO = group undergoing cycling exercise and maze training, Covered shift of attention = subtest ‘Covered Shift of Attention’ from TAP, CTR = passive controls, CVLT = ‘California Verbal Learning Test’, DST = ‘Digit Symbol Test’, ERGO = group undergoing cycling exercise, FRS = ‘Fragebogen Räumliche Strategien’ (questionnaire to assess spatial strategies), huWMZ = human analogue of the ‘Morris Water Maze’, IST = ‘Intelligence Structure Test 2000R’, MAZE = group undergoing maze training, RT = reaction time, VVM = ‘Test of Visual and Verbal Memory Retention’.

Longitudinal Change in Cognition: For cognitive tests, 11 of the 15 scores showed a significant effect of the linear term of time (p, uncorrected ≤ 0.028). Among them, the subscale ‘Global Navigation’ of a questionnaire assessing spatial strategies (‘Fragebogen Räumliche Strategien’ [FRS]^27^; FRS/global) as well as a component mainly reflecting reaction time (RT) and RT variability in the subtest ‘Alertness’ of ‘Tests of Attentional Performance’ (TAP)^28^ (Alertness A [RT/RT variability]) showed a random effect of time, indicated by a significant reduction in −2 log likelihood. However, factor group (ERGO/MAZE/COMBO/CTR) did not significantly interact with time (p, uncorrected ≥ 0.162), indicating that groups did not differ with regard to longitudinal change in cognitive performance. For the remaining cognitive performance scores, the interaction between group and time was not determined as models either revealed the absence of a significant fixed effect of time (p, uncorrected ≥ 0.086) or did not improve after adding a random effect of time, indicating the absence of linear within- and systematic between-subjects variance (see Fig. 3). Due to missing and excluded data, results were obtained for overall n ≥ 343 cases (≈ 87%).

**Figure 4:**
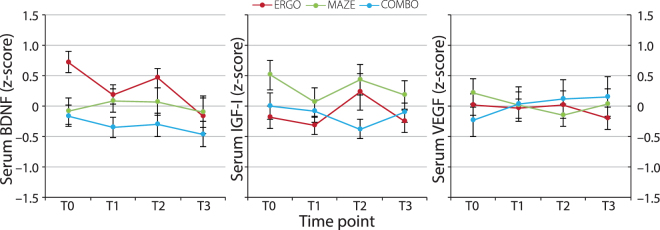
Group Means (± SEM) of Serum BDNF, IGF-I, and VEGF at Time Points T0–T3. Values represent z-scores, obtained across groups and time points after exclusion of outliers. Note that CTR did not take part in blood sampling. We did not observe group differences in linear change over time. BDNF = brain-derived neurotrophic factor, COMBO = group undergoing cycling exercise and maze training, CTR = passive controls, ERGO = group undergoing cycling exercise, IGF-I = insulin-like growth factor-I, MAZE = group undergoing maze training, VEGF = vascular endothelial growth factor.

Longitudinal Change in Growth Factors: Regarding growth factor levels, both IGF-I and VEGF revealed no significant fixed effect of the linear term of time (p, uncorrected ≥ 0.584), whereas BDNF showed a significant linear decrease over time (p, uncorrected = 0.005). Furthermore, the model for BDNF significantly improved after entering a random effect of time, suggesting substantial between-subjects variance in change over time. However, group (ERGO/MAZE/COMBO) did not significantly interact with time (p, uncorrected = 0.098), indicating the absence of differential transfer effects on longitudinal change in BDNF after different training regimes (see Fig. 4). Due to missing and excluded data, results were obtained for overall n ≥ 232 cases (≈ 79% of groups ERGO, MAZE, and COMBO).

**Figure 5:**
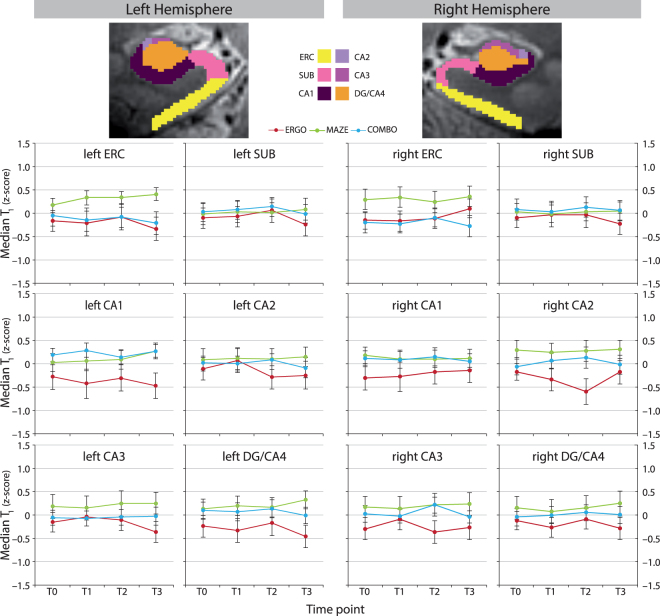
Group Means (± SEM) of Median T_1_ Relaxation Times of Left and Right Hippocampal Subfields at Time Points T0–T3. Values represent z-scores, obtained across groups and time points after exclusion of outliers. Note that CTR did not take part in 7 T MRI. We did not find evidence for systematic change over time. The upper panel shows the coronal view of an illustrative example of the manually delineated left and right hippocampal subfields, superimposed on a 7 T-TSE image. Overall, 12 hippocampi (6 left and 6 right) were manually delineated in order to provide atlases for subsequent automated delineations. Manual delineations were obtained based on coronal MR images acquired with the 7 T-TSE sequence at baseline T0. CA = cornu ammonis, COMBO = group undergoing cycling exercise and maze training, CTR = passive controls, DG = dentate gyrus, ERC = entorhinal cortex, ERGO = group undergoing cycling exercise, MAZE = group undergoing maze training, MRI = Magnetic Resonance Imaging, SUB = subiculum, TSE = turbo spin echo.

Longitudinal Change in Hippocampal Plasticity: Model testing for T_1_ relaxation times stopped after definition of baseline models due to the absence of significant fixed effects of linear time (p, uncorrected ≥ 0.186), indicating no systematic change over time after either training regime (see Fig. 5). Due to missing and excluded data, results were obtained for overall n ≥ 259 cases (≈ 87% of groups ERGO, MAZE, and COMBO).

Regarding our central research question, we conclude that irrespective of direct effects of the applied trainings, longitudinal transfer changes were comparable between the different experimental conditions.

#### Associations between Training-Induced Direct Change and Changes in Transfer Measures (Cognition, Growth Factors, and Hippocampal Plasticity)

Although training-induced effects on directly related performance metrics (i.e. PWC and navigation precision) did not expand to longitudinal transfer changes (i.e. cognition, growth factor levels, and hippocampal plasticity) within the study period of approximately 16 weeks, there might be associations between direct and transfer changes on a shorter time scale (i.e. changes from immediate pre- to immediate post-training). To test this assumption, we applied hierarchical regression analysis separately for the cycling exercise (collapsed over ERGO and COMBO) and maze training (collapsed over MAZE and COMBO). In other words, we analyzed whether and to what extent cycling gain (maze training gain, respectively) predicts transfer changes from immediate pre- to immediate post-cycling (pre- to post-maze, respectively) after correcting for covariates (baseline score of the criterion, initial age, and sex). Description of results is restricted to models that revealed significant change in R^2^ after entering cycling gain (maze training gain, respectively). For space reasons, we do not report covariates-only models.

Relationships between Cycling Gain and Transfer Changes from T0 (Pre-Cycling) to T1 (Post-Cycling): We observed a significantly positive correlation between cycling gain and change in using cardinal directions (FRS/cardinal directions; β = 0.362, ∆R^2^ = 0.130, p = 0.012). Additionally, change in FRS/cardinal directions correlated negatively with its baseline score (β = −0.432, p = 0.004). Neither initial age nor sex were associated with the criterion (β ≤ 0.167, p ≥ 0.241). Overall, the model explained 35.9% of variance (F_4,35_ = 4.898, p = 0.003). Furthermore, cycling gain showed a positive correlation with change in verbal memory retention as assessed with ‘Test of Visual and Verbal Memory Retention’ (VVM)^29,30^ (VVM A [verbal]; β = 0.337, ∆R^2^ = 0.111, p = 0.047). However, this finding should be treated with caution as the overall regression model did not reach statistical significance (R^2^ = 0.140, F_4,33_ = 1.340, p = 0.276; covariates: β ≤ −0.110, p ≥ 0.528). For the remaining transfer measures, covariates-only models did not significantly improve after entering cycling gain (p ≥ 0.079), indicating no further associations between cycling gain and transfer changes.

Relationships between Maze Training Gain and Transfer Changes from T1 (Pre-Maze) to T2 (Post-Maze): Hierarchical regression revealed a significantly positive correlation between maze training gain and change in the ‘Digit Symbol Test’ (DST)^31^, a test on processing speed (β = 0.306, ∆R^2^ = 0.088, p = 0.029). In this model (R^2^ = 0.328, F_4,39_ = 4.765, p = 0.003), change in DST was further related to its baseline score (β = −0.431, p = 0.004). Neither initial age nor sex were associated with the criterion (β ≤ −0.228, p ≥ 0.101). Furthermore, maze training gain was negatively related to change in IGF-I (β = −0.328, ∆R^2^ = 0.099, p = 0.026) as part of a model which explained 31.9% of variance (F_4,37_ = 4.326, p = 0.006). In this model, initial age revealed a negative correlation with change in IGF-I (β = −0.340, p = 0.021), whereas baseline score and sex did not reach statistical significance (β ≤ −0.289, p ≥ 0.052). For the remaining transfer measures, the amount of explained variance did not significantly increase after adding maze training gain to the model (p ≥ 0.062), indicating the absence of further associations between maze training gain and transfer changes.

## Discussion

In the present well-controlled study on a large sample of young, healthy volunteers, we observed substantial direct gain of both physical exercise and spatial training. This confirms the immediate effectiveness of either intervention. However, longitudinal change in various transfer domains, including cognitive performance, growth factor levels, and T_1_ relaxation times of hippocampal subfields, remained unaffected by both training regimes. Contrary what might be expected from animal studies, physical exercise did not augment progress in the subsequent spatial training. Evidence for an additive effect induced by a strictly sequential combination of physical exercise and cognitive stimulation comes from animal research^23^ and has been considered specifically for neurogenesis in the adult DG. Whether such an additive gain also applies to mechanisms other than adult neurogenesis and whether it transfers to the behavioral domain was not assessed. One explanation for our negative finding is that measurements in the present study were too coarse to capture the effect. Alternatively, the lack of an additive effect may indicate that physical exercise must be continued during the subsequent cognitive stimulation to elicit an additive effect^32^. Another explanation is that the spatial training used may have been insufficiently challenging to spur integration and persistence of new neurons into the hippocampal circuitry. Support for this view comes from an animal study showing that the morphological development of newly born hippocampal neurons is influenced by the level of cognitive demand induced by spatial learning^33^.

We did not observe cognitive transfer effects after physical exercise. This finding is at odds with past research that has suggested exercise-related cognitive improvement. Since a majority of previous studies investigated older adults, the present findings may indicate that the potential to induce training-related transfer changes is lesser in young, homogeneously well-educated adults. Indeed, results from a study in humans have been interpreted to show that baseline levels of adult neurogenesis may interact with the potential for change after physical exercise^34^. In that study, responders but not non-responders to exercise revealed an improvement in pattern separation. Since non-responders showed slightly greater levels of fitness and pattern separation performance at baseline, it may indicate that the performance change in the group of responders reflects performance normalization rather than improvement^34^. Similarly, in the present study the potential for change might have been reduced by relatively high baseline levels. In this vein, elderly people have been proposed to show a relatively greater potential for functional change as a consequence of age-associated neural dedifferentiation^35^. However, one has to keep in mind that our conclusions are based on results from a highly selective part of the overall German population. Generalizations of our findings to different human populations might therefore be limited.

We did not find training-related longitudinal change in growth factor levels. This may stem from the time course of training-induced change in growth factor levels. Training did not influence growth factor levels over sustained time periods, which is in line with other studies that have demonstrated a return to baseline within less than 1 h after cessation of training^36,37^. Regardless of the precise reason, the lack of evidence in the present study calls for a more precise definition of the role of growth factors regarding training effects on human brain structure and function. Interestingly, direct gain from spatial training correlated with change in IGF-I levels from immediate pre- to immediate post-maze, but did so in an inverse fashion.

Regarding cognitive transfer effects, we observed a positive correlation between gain from spatial training and change in digit symbol substitution. This is in line with previous findings that computerized cognitive training induces mild positive effects on various cognitive domains, including processing speed^38^. Cycling-induced change in PWC positively correlated with change in self-reported use of cardinal directions for spatial orientation. Furthermore, we observed a trend for a positive correlation between change in PWC and change in a cognitive component mainly reflecting verbal memory retention, a finding that is in line with previous research^6^. Past research has suggested that cognitive domains differentially respond to training. The ‘selective improvement’ hypothesis^39^, for example, states that exercise-induced effects on attention are restricted to tasks that require executive control processes and cognitive flexibility. Likewise, transfer effects on the memory domain were shown to require pattern separation^34^.

We did not observe transfer effects on longitudinal change in median T_1_ relaxation times of hippocampal subfields. Moreover, immediate pre- to immediate post-training change in T_1_ relaxation times did not correlate with either training gain. T_1_ is considered to mainly reflect myelination^25^, suggesting that our training paradigms did not change subfield myelination. An alternative explanation is that by analyzing median T_1_ relaxation times, we may not have captured focal change including neuro-, synapto-, and dendrogenesis with a less pronounced effect on myelination itself. In addition, automated delineation of hippocampal subfields *in vivo* as applied here might generally suffer from reduced reliability^40^ due to various aspects such as the small size of hippocampal subfields, between-subjects variability in hippocampal anatomy, resolution issues related to MRI and fusion of subfields in posterior parts of the hippocampus^41^.

## Methods

### Participants

99 volunteers aged 18 to 35 years were recruited in Leipzig, Germany. Participants were native German speakers or German speakers at a native level and indicated to be of normal weight, right-handed, and to have normal or corrected-to-normal vision. They had no history of psychiatric, neurological, cardiovascular, metabolic, or respiratory diseases. Further exclusion criteria were: regular intake of medication or drugs, pregnancy, and breastfeeding. Moreover, subjects who engaged in sport activity for more than 2.5 h per week were excluded from study participation. This exclusion criterion was meant to reduce baseline variance between participants as we expected exercise-related effects to vary with baseline fitness^42^. Moreover, by restricting the amount of sport activity, we aimed to prevent ceiling effects from obscuring the effectiveness of our cycling exercise. The cut-off value of 2.5 h per week was chosen based on practical considerations as, to our knowledge, there is no standard regarding the amount of competing sport activity. Furthermore, participants played first-person video games for a maximum of 1 h per week. This last exclusion criterion was based on three key assumptions: First, we wanted to keep groups as comparable as possible in terms of casual video game experience at baseline. Second, playing video games has been discussed to have broad effects on a number of cognitive functions (see ref.^43^ for an overview, or^44^, but also^45^ for a more critical evaluation) which might in turn lead to a training-related confound in cognitive performance measures. Third, former studies have reported influences of video game experience on learning performance (see ref.^46^ for video game-related influences on perceptual learning progress). Taken together, we therefore decided to restrict prior video game experience in our sample. The limit of 1 h per week was chosen both for practical reasons (to facilitate recruitment) and as this amount of experience per week is well below the inclusion criteria for video game players in former studies (e.g. ref.^46,47^). Consumption of nicotine or caffeine was not defined to be an exclusion criterion in order (i) to prevent the representativity of our sample from further declining and (ii) to facilitate recruitment of a sufficient sample. The proportion of smokers at baseline was balanced across groups (see Results), caffeine intake was not controlled for. All information was acquired during telephone screenings. MRI data collected during baseline or previous studies were evaluated by a physician. In case of brain abnormalities, participants were excluded. All procedures were carried out in accordance with the Declaration of Helsinki and were approved by the ethics committee of the Faculty of Medicine at the University of Leipzig (No. 164-13-03062013). Written informed consent was obtained from all participants before inclusion in the study.

### Study Design and Procedure

The study followed an experimental mixed design with time point (T0/T1/T2/T3) as a within-subjects factor and group (ERGO/MAZE/COMBO/CTR) as a between-subjects factor. Participants completed either eight 20-minute sessions of graded cycling based on high-intensity training between T0 and T1 (ERGO), sixteen 30-minute sessions of spatial training between T1 and T2 (MAZE), a sequential combination of both (COMBO), or they rested as passive controls (CTR; see Supplementary Information for training details). According to the training periods, T0 and T1 took place with an interval of approximately three weeks, whereas T1 and T2 took place with an interval of approximately five weeks. Time point T3 was implemented as non-intervention follow-up approximately seven weeks after T2. For groups ERGO, MAZE, and COMBO, each time point comprised blood sampling, 7 T MRI, and cognitive testing. Passive controls only attended the cognitive assessment with the aim of controlling for test-retest effects induced by repeated testing. To measure sustained rather than acute effects, post-intervention measurements took place approximately 1 to 2 days after the last training session. To take diurnal variations in growth factor levels^48^ into account, blood sampling was scheduled within limited morning slots (across subjects) and the within-subjects time of blood sampling was kept constant across sampling points within minor organizational constraints. Both cognitive testing and spatial training were scheduled throughout the day according to the individuals’ preferred time of day. For physical training, exercise slots were scheduled according to organizational constraints (availability of participants, trainer, medical background service, and exercise equipment) as the cycling exercise aimed at stimulating plastic changes in the human brain rather than inducing neuromuscular adaptations and diurnal variations have been demonstrated particularly for the latter (see ref.^49^ for review). Likewise, we did not expect the time of day to substantially affect MRI sessions as we measured brain structure rather than brain function.

#### Blood Sampling

Blood sampling took place in the morning between approximately 8:00 and 10:00 a.m. Participants were asked to avoid food intake for at least 2 h before. As far as possible, the sampling time was kept constant for each subject. Blood samples were briefly swayed and kept at room temperature for 30 min to then be centrifuged before serum was pipetted, aliquoted, and stored at −80 °C.

#### MR Image Acquisition

MRI data were collected on a Siemens MAGNETOM 7 T scanner (Siemens Healthineers, Erlangen, Germany) using a 24-channel head coil. We acquired whole-brain T_1_ maps using a magnetization-prepared two rapid acquisition gradient echoes (MP2RAGE) sequence^50^, recording 240 sagittal slices with anterior-posterior phase encoding direction providing a 0.7 mm isotropic resolution (repetition time TR = 5000 ms, echo time TE = 2.45 ms, inversion time TI_1/2_ = 900/2750 ms, flip angle FA_1/2_ = 5°/3°, field of view FOV = 224 × 224 mm, bandwidth BW = 250 Hz/Px, partial Fourier PF = 6/8). The acquisition was accelerated using GRAPPA with iPAT = 2. The total acquisition time (TA) for the MP2RAGE was 10:57 min. Furthermore, a turbo spin echo (TSE) sequence with a turbo factor of 8 was used to acquire 50 slices oriented perpendicular to the main axis of the hippocampus with inferior-superior phase encoding direction (TR = 16000 ms, TE = 14 ms, FA = 120°, FOV = 192 × 192 mm, voxel size = 0.5 × 0.5 × 1 mm, BW = 119 Hz/Px, TA = 13:06 min). No partial Fourier or parallel imaging was used for the TSE. Distortions caused by gradient non-linearities were corrected for using a gradient coil specific look-up table.

#### Cognitive Assessment

Cognitive performance was assessed by applying the following tests (German version, respectively): FRS, ‘Dresden Spatial Navigation Task’, which denotes a human analogue of the ‘Morris Water Maze’ (huWMZ), subtest ‘Location Memory’ from ‘Berlin Intelligence Structure Test’ (BIS)^51^, VVM, subtests ‘Figures’, ‘Dices’, and ‘Matrices’ from ‘Intelligence Structure Test 2000R’ (IST)^52^ as well as ‘California Verbal Learning Test’ (CVLT)^53^. By using these tests, we aimed to assess cognitive functions associated with the hippocampus, including memory performance (BIS, VVM, and CVLT) and spatial cognition (huWMZ, FRS, and IST). Since previous studies linked physical exercise to improved attention and processing speed^1^, we additionally applied subtests ‘Alertness’ and ‘Covered Shift of Attention’ from TAP as well as the DST. To minimize ceiling effects in both the DST and CVLT, we slightly modified the test procedure, respectively. For the DST, we reduced the time limit from 90 to 60 s. For the CVLT, we performed three instead of five learning trials and extended the wordlists of version 1 and 2 by adding words from version 3. Therefore, we did not use standard scores provided by the test manuals.

### Data Analysis

#### Preprocessing

Cycling Exercise: Cycling gain was determined by calculating percent change in PWC120, PWC150, and PWC170, respectively, with PWC being operationally defined by measuring pedal resistance in watts (W) at predefined mean heart rates of 120, 150, and 170 bpm^54^. For a few subjects, we had to substitute PWC170 by using PWC at the maximum mean heart rate as they did not reach a mean heart rate of 170 bpm. To control for differences in physical constitution, PWC values were divided by body weight at baseline T0.

Maze Training: For the maze training, we estimated the AUC for session performance over session number by calculating the sum of performance averages between consecutive sessions. Session performance was defined by respectively determining navigation precision for the most difficult, yet successfully completed level. To obtain navigation precision, we multiplied the optimal path length of this level by its difficulty and divided the resulting product by the mean actual path length. This calculation was based on theoretical considerations so that higher values indicate greater navigation precision. Path length was chosen to be the variable of interest in order to get a measure that is sensitive to both (i) random navigation behavior (e.g. always going left at crossings) and (ii) false navigation decisions.

Cognitive Test Data: Due to the large number of cognitive variables, we reduced the initial data set to a smaller size by applying principal component analysis (PCA) separately for each cognitive test with two or more output variables. To increase the subjects-to-variable ratio, PCAs were applied to the entire data set after within-group and -time z-transformation. Standardization was done after exclusion of outliers, resulting in n ≥ 343 cases. We used parallel analysis^55^ to determine the number of components. The oblimin (oblique) method was chosen for rotating components. PCAs led to 10 components: huWMZ/path length, IST/figural, VVM A (verbal), VVM B (visual forgetting rate), VVM C (visual), modified CVLT, Alertness A (RT/RT variability), Alertness B (phasic shift), Covered shift of attention A (RT/RT variability), and Covered shift of attention B (valid shift). Component scores were obtained according to the component score coefficient matrix (see Supplementary Table S1) after re-calculation of z-scores across groups and time points.

Blood Samples: Serum levels of BDNF, IGF-I, and VEGF were determined using Enzyme-linked Immunosorbent Assay (ELISA) kits (R&D SYSTEMS, Wiesbaden, Germany) according to the manufacturer’s instruction. When necessary, samples were diluted to fit the measurement ranges of the ELISA kits. The intra- and inter-assay coefficients were 4.2% and 6.5% for VEGF, 6.1% and 8.9% for BDNF, and 7.9% and 10.7% for IGF-I.

**Figure 6:**
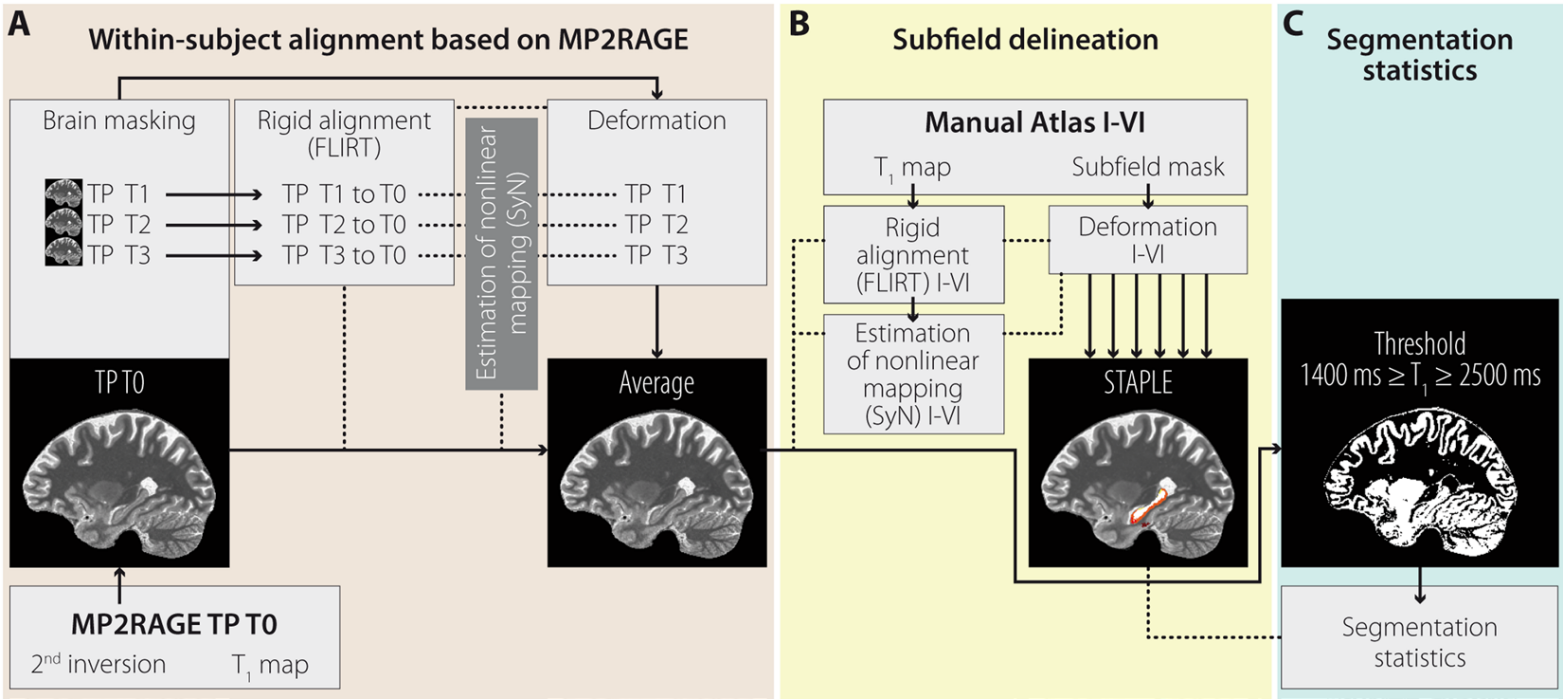
Preprocessing of the T_1_ Map Acquired with 7 T MP2RAGE. Preprocessing included **(A)** skull stripping and within-subject alignment of the T_1_ maps, **(B)** automated delineation of hippocampal subfields, and **(C)** estimation of median T_1_ relaxation time for each hippocampal subfield. Note that within-subject alignment of time points T1, T2, and T3 is not shown as it resembled the procedure for time point T0, respectively. MR images refer to an exemplary sagittal view of a single subject for illustrative purposes. FLIRT = FMRIB’s Linear Image Registration Tool, MP2RAGE = magnetization-prepared two rapid acquisition gradient echoes, MR = Magnetic Resonance, STAPLE = simultaneous truth and performance level estimation, SyN = symmetric normalization method, TP = time point.

7 T MRI Data: MR Image preprocessing was done using CBSTools^56^ and tools from MIPAV^57^, JIST^58^, and ANTs^59^ integrated into an automated JIST processing pipeline. First, we obtained brain masks for each subject and time point based on the second inversion and T_1_ map acquired with MP2RAGE. A description of the skull stripping method can be found elsewhere^56^. Next, time points were mapped to each other based on MIPAV’s implementation of the FMRIB’s Linear Image Registration Tool (FLIRT)^60–62^ for rigid alignment followed by nonlinear deformations estimated with the symmetric normalization method (SyN) from the ANTs package. Hippocampal subfields were automatically delineated using simultaneous truth and performance level estimation (STAPLE)^63^, which estimates a probabilistic true segmentation of hippocampal subfields based on the combination of multiple atlases. Atlases were obtained through manual subfield delineation in six subjects (each left and right hippocampus) using coronal slices of baseline TSE and a full-length procedure^41^. Subfields included ERC, SUB, CA1, CA2, CA3, and DG/CA4 (see Fig. 5). We used version 2.2.0 of ITK-SNAP^64^ for manual delineations. Atlases were then mapped to the within-subject averages of each individual subject and time point via non-linear deformation with SyN. To prevent insufficient accuracy of boundary delineation to affect T_1_ estimation, we created a binary mask for the T_1_ map according to a range of 1400 ≤ T_1_ ≤ 2500 ms (see Fig. 6). In addition, we analyzed median T_1_ rather than mean T_1_.

#### Statistical Analysis

Exclusion of Outliers: Outliers were excluded using the outlier labeling rule with a factor g of 2.2^65,66^. To this end, percentiles were calculated across groups and time points. Supplementary Table S2 summarizes the number of excluded cases for each variable of interest.

Direct Effects of the Applied Training Regimes: 

Both cycling and maze training gain were analyzed by applying one-sample t-tests after exclusion of outliers. The critical test value was set to zero, respectively. Whereas cycling gain was collapsed over ERGO and COMBO, maze training gain was analyzed separately for MAZE and COMBO according to our main hypothesis. Group differences in maze training gain were examined using an independent-samples t-test. Alpha levels were set to 5%.

Effects on Longitudinal Transfer Changes: 

To examine group differences in longitudinal transfer changes, we applied linear mixed modeling according to a multistep procedure (method: maximum likelihood)^67^. By using linear mixed models, we were able to control for both between-subjects differences in the number of days since baseline T0 (see Table 2) and missing data (see Supplementary Table S2). Models were defined separately for each variable of interest, including 15 cognitive performance scores, serum BDNF, IGF-I and VEGF, and median T_1_ relaxation times of 12 hippocampal subfields. Longitudinal change was modeled from T0 to T3 across all groups (except CTR for growth factors and T_1_ relaxation times), respectively. In a first step, we added the fixed effect of linear time to the fixed and random intercept. If the fixed effect of linear time reached significance, we assessed whether model fit was improved by the random effect of linear time, indicated by a significant reduction in −2 log likelihood. In case of a random effect of linear time, we determined the change in model fit after adding the (unstructured) covariance between random intercept and random time. Then, our predictors of interest (group and group × time) and covariates (age, sex, age × time, and sex × time) were entered. Dependent variables except cognitive component scores, time (days), and age were z-standardized across groups and time points after exclusion of outliers (see Supplementary Table S3 for raw scores).

**Table 2:** Mean Number of Days (Min, Max) between Baseline (T0) and Follow-Ups (T1, T2, and T3).

	CTR	ERGO	MAZE	COMBO	p
∆_T0,T1_	21.35 (20, 24)	20.41 (16, 22)	20.91 (17, 24)	20.83 (17, 23)	0.029
∆_T0,T2_	55.88 (46, 61)	59.59 (53, 79)	55.74 (52, 60)	55.83 (51, 64)	0.007
∆_T0,T3_	106.57 (104, 133)	105.80 (95, 113)	105.23 (100, 118)	104.91 (100, 111)	0.553

Values refer to the time points of cognitive assessment, respectively. We obtained p-values by applying analysis of variance (ANOVA). To account for between-subjects differences in the number of days since baseline T0, we applied linear mixed modeling to analyze longitudinal changes. COMBO = group undergoing cycling exercise and maze training, CTR = passive controls, ERGO = group undergoing cycling exercise, MAZE = group undergoing maze training.

Associations between Training-Induced Direct Change and Changes in Transfer Measures (Cognition, Growth Factors, and Hippocampal Plasticity): Furthermore, we analyzed whether the direct gain induced by cycling (collapsed over ERGO and COMBO) and spatial training (collapsed over MAZE and COMBO), respectively, correlated with transfer changes by applying hierarchical regression (method: enter, listwise exclusion of missing data). For this analysis, cycling gain was defined by averaging percent change in PWC120, PWC150, and PWC170. Maze training gain was obtained by estimating the AUC for navigation precision over session number. Transfer change was determined by calculating percent change from immediate pre- to immediate post-intervention based on raw scores. For cognitive components, we defined differences. Thus, different time points were considered for the cycling exercise (T0 vs. T1) and maze training (T1 vs. T2). In a first step, we defined a model with covariates (baseline score of the criterion, initial age, and sex) entered as predictors. Second, we added cycling gain (maze training gain, respectively) to the list of predictors. Subsequent significant change in the amount of explained variance indicated a substantial relationship between direct gain and transfer change. Results were obtained after exclusion of cases with a standardized residual greater than ± 2 or a Cook’s distance greater than 1, resulting in n ≥ 32 subjects. Variance inflation factor (VIF) scores were less than 1.61, revealing the absence of multicollinearity. We report uncorrected p-values. We used version 24 of IBM SPSS Statistics for statistical analysis.

### Data availability

The data sets generated during the current study are available in anonymized form from the corresponding authors on reasonable request.

## Electronic supplementary material

Supplementary Information
