# Supplementary material for: Physical Exercise and Spatial Training: A Longitudinal Study of Effects on Cognition, Growth Factors, and Hippocampal Plasticity
Source: Sci Rep. 2018 Mar 9;8:4239. doi: 10.1038/s41598-018-19993-9 (PMC5844866; doi:10.1038/s41598-018-19993-9)
Supplement: Supplementary file 1 — Supplementary Information [file 41598_2018_19993_MOESM1_ESM.pdf]

Supplementary Information for

Physical Exercise and Spatial Training: A Longitudinal Study of Effects on Cognition, Growth Factors, and Hippocampal Plasticity

Luise Woost, Pierre-Louis Bazin, Marco Taubert, Robert Trampel, Christine L. Tardif, Alexander Garthe, Gerd Kempermann, Ulrich Renner, Günter Stalla, Derek V. M. Ott, Viola Rjosk, Hellmuth Obrig, Arno Villringer, Elisabeth Roggenhofer, Tilmann A. Klein

Training Interventions

Physical (Cycling) Exercise

The physical exercise consisted of eight supervised 20-minute sessions of cycling on a stationary cycle ergometer. For all participants taking part in the physical exercise, cycling sessions were scheduled on separate days and distributed as equally as possible between time points T0 and T1. Across groups ERGO and COMBO, there was an average inter-session interval of 2.27 (± 1.47) days (ERGO: M = 2.26, SD = 1.55; COMBO: M = 2.29, SD = 1.39). During exercise, participants' heart rate was monitored using Polar RS400 and Polar ProTrainer 5 (Polar Electro Oy, Kempele, Finland). Subjects were asked to cycle at 60 resolutions per minute (rpm). In sessions 1 and 8, we conducted graded fitness assessments in order to determine participants' individual physical working capacity (PWC). PWC was defined by measuring pedal resistance in watts (W) at predefined heart rates of 120 (PWC120), 150 (PWC150), and 170 bpm (PWC170). During both assessment sessions, participants started to cycle at 25 W. At 2-minute intervals, the pedal resistance was continuously increased by further 25 W until participants showed a target heart rate of 170 bpm. In case participants did not show a heart rate of 170 bpm, the maximum heart rate was considered instead. In sessions 2 to 7, the exercise intensity was gradually changed to vary between individual pre-cycling PWC120 and pre-cycling PWC170 using KETTLER WORLD TOURS 2.0 (KETTLER GmbH, Ense-Parsit, Germany). To account for potential fitness adaptations in response to the exercise, the cycling intensity of sessions 5 to 7 was slightly increased. Figure S1 provides details of either training scheme.

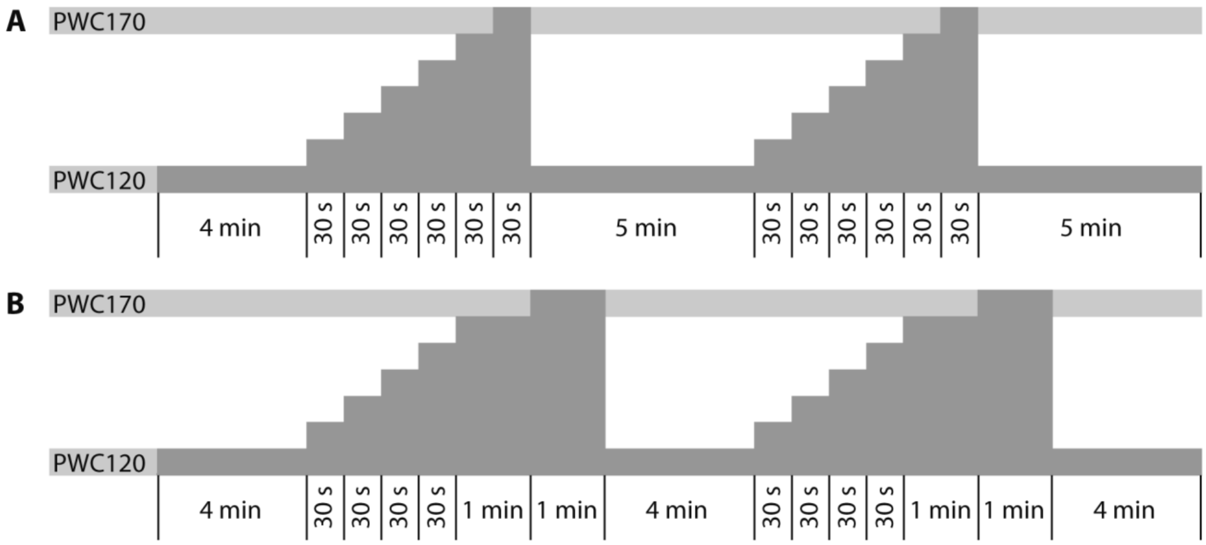

Supplementary Figure S1. Cycling scheme for **(A)** exercise sessions 2 to 4 and **(B)** exercise sessions 5 to 7. In sessions 1 and 8, we conducted graded fitness assessments (not shown). bpm = beats per minute, PWC120 = physical working capacity at 120 bpm, PWC170 = physical working capacity at 170 bpm.

Spatial (Maze) Training

Spatial training comprised sixteen 30-minute sessions of a computerized maze learning task. For all participants taking part in the maze training, training sessions were scheduled on separate days and distributed as equally as possible between time points T1 and T2. Across groups MAZE and COMBO, there was an average inter-session interval of 2.03 (± 1.30) days (MAZE: M = 2.05, SD = 1.26; COMBO: M = 2.01, SD = 1.33). Participants were asked to navigate to the exit of a maze via the most direct route, thus without going into dead ends. Mazes were presented through a first-person perspective on a standard computer screen. Mazes differed with regard to both the number of crossings and the time limit, resulting in various difficulty levels. Mazes were presented in an adaptive manner: As soon as participants solved a maze (i.e. found the exit within the time limit) three times in a row, they entered the next level. In case participants failed to solve a maze (i.e. time limit expired without finding the exit) three times in a row, they returned to the previous level. To prompt an allocentric spatial reference frame, mazes were surrounded by concentrically arranged buildings and trees.

Supplementary Table S1

Calculation of Principal Components according to the Component Score Coefficient Matrix

Component	Calculation
huWMZ/path length	(.578*zhuWMZ_path_AUC) + (.578*zhuWMZ_path_probe)
VVM A (verbal)	(-.011*zVVM_visual_immediate) + (-.004*zVVM_visual_delayed) + (.018*zVVM_visual_forgetting) + (.451*zVVM_verbal_immediate) + (.477*zVVM_verbal_delayed) + (.223*zVVM_verbal_forgetting)
VVM B (visual forgetting rate)	(-.234*zVVM_visual_immediate) + (.250*zVVM_visual_delayed) + (.756*zVVM_visual_forgetting) + (.094*zVVM_verbal_immediate) + (-.034*zVVM_verbal_delayed) + (-.331*zVVM_verbal_forgetting)
VVM C (visual)	(-1)*([-.564*zVVM_visual_immediate] + [-.491*zVVM_visual_delayed] + [.020*zVVM_visual_forgetting] + [.050*zVVM_verbal_immediate] + [.003*zVVM_verbal_delayed] + [-.104*zVVM_verbal_forgetting])
IST/figural	(.449*zIST_figures) + (.455*zIST_dices) + (.401*zIST_matrices)
Modified CVLT	(.303*zCVLT_sum) + (.302*zCVLT_VWI) + (.297*zCVLT_VWII) + (.228*zCVLT_B)
Alertness A (RT/RT variability)	(.329*zALMDNton) + (.235*zALSTDton) + (.363*zALMDNphas) + (.301*zALSTDphas) + (-.027*zALphasic_shift)
Alertness B (phasic shift)	(.186*zALMDNton) + (.234*zALSTDton) + (-.181*zALMDNphas) + (-.163*zALSTDphas) + (.844*zAlphas_shift)
Covered shift of attention A (RT/RT variability)	(.323*zCSMDNvalid) + (.390*zCSSTDvalid) + (.286*zCSMDNinval) + (.290*zCSSTDinval) + (-.118*zCS_Fval)
Covered shift of attention B (valid shift)	(.138 *zCSMDNvalid) + (-.226*zCSSTDvalid) + (.381*zCSMDNinval) + (-.182*zCSSTDinval) + (.707*zCS_Fval)

Note. Components were obtained using principal component analysis (PCA; see Methods). Component scores were determined based on z-scores, z-scores were obtained across groups and time points. Prior to z-standardization, outliers were excluded using the outlier labeling rule (see Methods). Note that we modified the CVLT (see Methods). Alertness = subtest 'Alertness' from 'Tests of Attentional Performance' (TAP), ALMDNphas = median reaction time in phasic trials of TAP-subtest 'Alertness', ALMDNton = median reaction time in tonic trials of TAP-subtest 'Alertness', ALphasic_shift = difference of median reaction time in tonic trials vs. median reaction time in phasic trials of TAP-subtest 'Alertness', ALSTDphas = standard deviation of reaction time in phasic trials of TAP-subtest 'Alertness', ALSTDton = standard deviation of reaction time in tonic trials of TAP-subtest 'Alertness', Covered shift of attention = subtest 'Covered Shift of Attention' from TAP, CS_Fval = main effect of cue validity in TAP-subtest 'Covered Shift of Attention', CSMDNinval = median reaction time in invalid-cue trials of TAP-subtest 'Covered Shift of Attention', CSMDNvalid = median reaction time in valid-cue trials of TAP-subtest 'Covered Shift of Attention', CSSTDinval = standard deviation of reaction time in invalid-cue trials of TAP-subtest 'Covered Shift of Attention', CSSTDvalid = standard deviation of reaction time in valid-cue trials of TAP-subtest 'Covered Shift of Attention', CVLT = 'California Verbal Learning Test', CVLT_B = immediate recall of wordlist B in CVLT, CVLT_sum = learning sum in CVLT, CVLT_VWI = free recall I in CVLT, CVLT_VWII = free recall II in CVLT, huWMZ = human analogue of the 'Morris Water Maze', huWMZ_path_AUC = area under the curve (AUC)

for path length over 6 trials of huWMZ, huWMZ_path_probe = path length in probe trial of huWMZ, IST = ‘Intelligence Structure Test 2000R’, IST_dices = IST-subtest ‘Dices’, IST_figures = IST-subtest ‘Figures’, IST_matrices = IST-subtest ‘Matrices’, RT = reaction time, VVM = ‘Test of Visual and Verbal Memory Retention’, VVM_verbal_delayed = delayed verbal recall in VVM, VVM_verbal_forgetting = verbal forgetting rate in VVM, VVM_verbal_immediate = immediate verbal recall in VVM, VVM_visual_delayed = delayed visual recall in VVM, VVM_visual_forgetting = visual forgetting rate in VVM, VVM_visual_immediate = immediate visual recall in VVM.

Supplementary Table S2

Outliers and Missing Data

Variable	Group	Outlier cases				Missing cases				
		Number				Number				Reason
$AUC_{navigation\ precision}$	MAZE					1				No AUC calculated because of missing performance score for one session (2)
	COMBO					1				
Pre- to post-cycling change (%) in PWC120	ERGO	2				3				Loss of pre-cycling PWC data due to technical reasons (4)
	COMBO					1				
Pre- to post-cycling change (%) in PWC150	ERGO					3				Loss of pre-cycling PWC data due to technical reasons (4)
	COMBO					1				
Pre- to post-cycling change (%) in PWC170	ERGO					3				Loss of pre-cycling PWC data due to technical reasons (4)
	COMBO					1				
		Time point				Time point				
		T0	T1	T2	T3	T0	T1	T2	T3	
BDNF	ERGO			1			4	4	6	Time point cancelled due to technical issues with the MRI scanner (13), pregnancy (1), or personal reasons (2); food intake shortly before blood sampling (4)
	MAZE	1				1		1	2	
	COMBO							1	1	
IGF-I	ERGO	1		1	1		4	4	6	Time point cancelled due to technical issues with the MRI scanner (13), pregnancy (1), or personal reasons (2); food intake shortly before blood sampling (4)
	MAZE	2	2	2	2	1		1	2	
	COMBO							1	1	
VEGF	ERGO			1		2	5	6	6	Time point cancelled due to technical issues with the MRI scanner (13), pregnancy (1), or personal reasons (2); food intake shortly before blood sampling (4); values were below the detection limit (5 pg/ml; 33)
	MAZE		1		1	3	1	2	4	
	COMBO	2		2		5	6	7	6	

FRS/global	CTR								1	3	Time point cancelled due to technical issues with the MRI scanner (13), pregnancy (1), or personal reasons (6); missing answers (3)
	ERGO							4	5	7	
	MAZE									2	
	COMBO									1	
FRS/allocentric	CTR								1	3	Time point cancelled due to technical issues with the MRI scanner (13), pregnancy (1), or personal reasons (6); missing answers (5)
	ERGO							4	4	8	
	MAZE								1	2	
	COMBO								1	1	
FRS/cardinal directions	CTR							1	2	3	Time point cancelled due to technical issues with the MRI scanner (13), pregnancy (1), or personal reasons (6); missing answers (3)
	ERGO							5	4	6	
	MAZE									1	
	COMBO									1	
Modified DST	CTR								1	3	Time point cancelled due to technical issues with the MRI scanner (13), pregnancy (1), or personal reasons (6)
	ERGO							4	4	6	
	MAZE									1	
	COMBO									1	
BIS/location memory	CTR								1	3	Time point cancelled due to technical issues with the MRI scanner (13), pregnancy (1), or personal reasons (6)
	ERGO							4	4	6	
	MAZE									1	
	COMBO									1	
huWMZ/path length	CTR						1		1	3	Time point cancelled due to technical issues with the MRI scanner (13), pregnancy (1), or personal reasons (6); probe trial of huWMZ not completed (1)
	ERGO							4	4	6	
	MAZE									1	
	COMBO									1	
IST/figural	CTR						1	1	2	4	Time point cancelled due to technical issues with the MRI scanner (13), pregnancy (1), or personal reasons (6); IST-subtest ‘Matrices’ not performed (4)
	ERGO							4	4	6	
	MAZE									1	
	COMBO									1	
VVM A (verbal)	CTR	1	1	1					1	3	Time point cancelled due to technical issues with the MRI scanner (13), pregnancy (1), or personal reasons (6)
	ERGO	2		1				4	4	6	
	MAZE	2	2	1	2					1	

	COMBO	1		1						1	
VVM B (visual forgetting rate)	CTR	1	1	1					1	3	Time point cancelled due to technical issues with the MRI scanner (13), pregnancy (1), or personal reasons (6)
	ERGO	2		1				4	4	6	
	MAZE	2	2	1	2					1	
	COMBO	1		1						1	
VVM C (visual)	CTR	1	1	1					1	3	Time point cancelled due to technical issues with the MRI scanner (13), pregnancy (1), or personal reasons (6)
	ERGO	2		1				4	4	6	
	MAZE	2	2	1	2					1	
	COMBO	1		1						1	
Modified CVLT	CTR								1	3	Time point cancelled due to technical issues with the MRI scanner (13), pregnancy (1), or personal reasons (6)
	ERGO							4	4	6	
	MAZE									1	
	COMBO									1	
Alertness A (RT/RT variability)	CTR	3	1	1	2				1	3	Time point cancelled due to technical issues with the MRI scanner (13), pregnancy (1), or personal reasons (6); data loss due to technical reasons (1)
	ERGO	1	1		2			4	4	6	
	MAZE	3	1	1	2			1		1	
	COMBO	2	1	1	1					1	
Alertness B (phasic shift)	CTR	3	1	1	2				1	3	Time point cancelled due to technical issues with the MRI scanner (13), pregnancy (1), or personal reasons (6); data loss due to technical reasons (1)
	ERGO	2	1		2			4	4	6	
	MAZE	3	2	1	3			1		1	
	COMBO	2	1	1	1					1	
Covered shift of attention A (RT/RT variability)	CTR	2	1	2	2				1	3	Time point cancelled due to technical issues with the MRI scanner (13), pregnancy (1), or personal reasons (6); data loss due to technical reasons (3)
	ERGO	1	3	1	2			4	4	6	
	MAZE	2	3	2	2			1	1	1	
	COMBO	2	1	2	2		1			1	
Covered shift of attention B (valid shift)	CTR	2	1	2	2				1	3	Time point cancelled due to technical issues with the MRI scanner (13), pregnancy (1), or personal reasons (6); data loss due to technical reasons (3)
	ERGO	1	3	1	2			4	4	6	
	MAZE	2	3	2	2			1	1	1	
	COMBO	2	1	2	2		1			1	
T_1 relaxation time ERC left/right	ERGO						2/2	6/6	6/6	8/8	Time point cancelled due to technical issues with the MRI scanner (12), pregnancy (1), MRI exclusion criterion (1), or personal reasons (2); no reliable
	MAZE						1/1	1/1	1/1	2/2	

	COMBO	1/0	1/0	1/0	1/0			1/1	1/1	subfield mask obtained (12); data loss due to technical reasons (1)
T_1 relaxation time	ERGO					2/2	6/6	6/6	8/8	Time point cancelled due to technical issues with the MRI scanner (12),
SUB left/right	MAZE	0/1	0/1	0/1	0/1	1/1	1/1	1/1	2/2	pregnancy (1), MRI exclusion criterion (1), or personal reasons (2); no reliable
	COMBO							1/1	1/1	subfield mask obtained (12); data loss due to technical reasons (1)
T_1 relaxation time	ERGO			0/1	0/1	2/2	6/6	6/6	8/8	Time point cancelled due to technical issues with the MRI scanner (12),
CA1 left/right	MAZE					1/1	1/1	1/1	2/2	pregnancy (1), MRI exclusion criterion (1), or personal reasons (2); no reliable
	COMBO							1/1	1/1	subfield mask obtained (12); data loss due to technical reasons (1)
T_1 relaxation time	ERGO	0/1				2/2	6/6	6/6	8/8	Time point cancelled due to technical issues with the MRI scanner (12),
CA2 left/right	MAZE					1/1	1/1	1/1	2/2	pregnancy (1), MRI exclusion criterion (1), or personal reasons (2); no reliable
	COMBO	1/0	1/0	1/0				1/1	1/1	subfield mask obtained (12); data loss due to technical reasons (1)
T_1 relaxation time	ERGO					2/2	6/6	6/6	8/8	Time point cancelled due to technical issues with the MRI scanner (12),
CA3 left/right	MAZE					1/1	1/1	1/1	2/2	pregnancy (1), MRI exclusion criterion (1), or personal reasons (2); no reliable
	COMBO							1/1	1/1	subfield mask obtained (12); data loss due to technical reasons (1)
T_1 relaxation time	ERGO	0/1	0/1	0/1	0/1	2/2	6/6	6/6	8/8	Time point cancelled due to technical issues with the MRI scanner (12),
DG/CA4 left/right	MAZE					1/1	1/1	1/1	2/2	pregnancy (1), MRI exclusion criterion (1), or personal reasons (2); no reliable
	COMBO							1/1	1/1	subfield mask obtained (12); data loss due to technical reasons (1)

Note. Outliers were excluded using the outlier labeling rule (see Methods). Note that CTR only attended the cognitive assessment in order to control for test-retest effects induced by repeated testing. Alertness = subtest 'Alertness' from 'Tests of Attentional Performance' (TAP), AUC = area under the curve, BDNF = brain-derived neurotrophic factor, BIS = 'Berlin Intelligence Structure Test', CA = cornu ammonis, COMBO = group undergoing cycling exercise and maze training, Covered shift of attention = subtest 'Covered Shift of Attention' from TAP, CTR = passive controls, CVLT = 'California Verbal Learning Test', DG = dentate gyrus, DST = 'Digit Symbol Test', ERC = entorhinal cortex, ERGO = group undergoing cycling exercise, FRS = 'Fragebogen Räumliche Strategien' (questionnaire to assess spatial strategies), huWMZ = human analogue of the 'Morris Water Maze', IGF-I = insulin-like growth factor-I, IST = 'Intelligence Structure Test 2000R', MAZE = group undergoing maze training, MRI = magnetic resonance imaging, PWC120 = physical working capacity at 120 bpm, PWC150 = physical working capacity at 150 bpm, PWC170 = physical working capacity at 170 bpm, RT = reaction time, SUB = subiculum, VEGF = vascular endothelial growth factor, VVM = 'Test of Visual and Verbal Memory Retention'.

Supplementary Table S3

Unstandardized Group Means (SD) of Selected Transfer Measures at Time Points T0 – T3

Variable		Group CTR				Group ERGO				Group MAZE				Group COMBO			
		T0	T1	T2	T3	T0	T1	T2	T3	T0	T1	T2	T3	T0	T1	T2	T3
FRS/global	M	4.3	4.2	4.1	4.0	4.6	4.3	4.2	4.2	4.1	4.1	4.0	3.8	4.2	4.1	4.1	4.2
	SD	1.0	1.1	1.1	1.2	1.1	1.2	1.2	1.2	1.2	1.3	1.3	1.3	1.2	1.2	1.2	1.2
FRS/allocentric	M	3.8	3.6	3.7	3.5	4.0	4.1	4.1	3.8	3.6	3.7	3.5	3.5	4.1	3.7	3.4	3.8
	SD	1.0	1.2	1.2	1.1	1.2	1.1	1.3	1.3	1.4	1.4	1.3	1.4	1.1	1.5	1.5	1.4
FRS/cardinal	M	3.1	3.1	3.3	3.1	3.3	3.3	3.3	3.2	2.8	2.8	3.1	3.1	3.3	3.6	3.4	3.8
directions	SD	1.6	1.4	1.3	1.7	1.3	1.3	1.3	1.7	1.4	1.5	1.7	1.5	1.7	1.7	1.6	1.8
Modified DST	M	44.2	46.8	49.8	51.3	45.8	48.8	49.8	50.4	48.1	49.7	52.7	52.7	45.0	49.5	52.3	53.2
	SD	5.3	5.3	5.1	5.6	5.6	6.1	8.0	6.3	7.2	7.0	7.8	7.1	5.5	7.4	6.5	8.1
BIS/location	M	17.5	19.8	19.4	19.9	19.1	20.4	19.4	21.6	17.9	21.3	21.9	21.1	18.0	21.4	21.5	22.0
memory	SD	4.5	4.5	4.6	5.5	4.4	4.1	3.6	3.2	4.3	4.5	3.5	3.7	4.1	3.3	3.2	3.7
BDNF	M	-	-	-	-	21.9	19.6	20.8	18.0	18.3	19.1	19.0	18.3	18.0	17.1	17.3	16.6
	SD					4.0	3.5	3.4	5.8	4.5	4.2	4.8	5.4	3.9	3.8	4.3	4.6
IGF-I	M	-	-	-	-	1,845.5	1,677.4	2,325.5	1,766.0	2,651.6	2,126.2	2,558.4	2,262.3	2,055.5	1,960.8	1,612.5	1,930.4
	SD					1,065.8	822.8	1,615.9	944.0	1,272.7	1,305.1	1,317.5	1,194.4	1,247.5	1,025.0	881.3	913.4
VEGF	M	-	-	-	-	30.9	29.3	30.8	25.6	35.3	30.7	26.7	31.3	24.9	31.1	33.0	33.8
	SD					19.3	17.7	22.7	19.3	25.0	23.0	19.5	24.1	25.3	28.6	28.8	32.3
T_1 relaxation time	M	-	-	-	-	2,164.6	2,162.5	2,167.5	2,158.3	2,176.1	2,181.8	2,181.8	2,184.5	2,168.5	2,165.2	2,167.0	2,162.5
ERC left	SD					39.0	41.8	44.5	37.4	25.0	25.8	22.1	21.6	37.9	41.1	35.7	40.6
T_1 relaxation time	M	-	-	-	-	1,985.4	1,987.5	1,996.2	1,976.4	1,990.9	1,994.3	1,993.2	1,997.6	1,993.7	1,996.9	2,001.1	1,991.3
SUB left	SD					69.9	62.6	72.7	65.6	69.2	67.2	64.6	68.9	64.0	64.8	65.1	62.4
T_1 relaxation time	M	-	-	-		2,089.6	2,083.7	2,088.7	2,081.9	2,102.3	2,103.4	2,104.5	2,111.9	2,108.3	2,112.5	2,106.5	2,112.0

CA1 left	SD					52.1	56.9	50.3	46.8	36.1	38.0	39.1	31.2	28.2	29.5	27.4	27.0
T_1 relaxation time	M	-	-	-	-	**2,120.8**	**2,135.0**	**2,106.2**	**2,109.7**	**2,136.4**	**2,138.6**	**2,137.5**	**2,141.7**	**2,131.5**	**2,129.3**	**2,136.4**	**2,122.8**
CA2 left	SD					92.0	89.4	88.1	99.3	89.2	87.5	85.5	78.4	55.5	61.5	50.4	81.5
T_1 relaxation time	M	-	-	-	-	**2,056.2**	**2,062.5**	**2,058.7**	**2,044.4**	**2,075.0**	**2,072.7**	**2,078.4**	**2,078.6**	**2,061.5**	**2,060.4**	**2,062.0**	**2,063.0**
CA3 left	SD					57.2	47.6	56.9	53.2	66.8	66.8	70.0	58.8	43.0	42.3	45.1	49.9
T_1 relaxation time	M	-	-	-	-	**2,263.5**	**2,260.0**	**2,266.2**	**2,255.6**	**2,277.3**	**2,279.5**	**2,278.4**	**2,284.5**	**2,276.0**	**2,275.0**	**2,277.2**	**2,271.7**
DG/CA4 left	SD					41.7	42.5	46.1	37.9	35.3	35.9	36.4	32.1	33.4	33.8	33.6	31.4
T_1 relaxation time	M	-	-	-	-	**2,164.6**	**2,163.7**	**2,166.2**	**2,176.4**	**2,185.2**	**2,187.5**	**2,183.0**	**2,188.1**	**2,162.5**	**2,161.5**	**2,167.4**	**2,158.7**
ERC right	SD					41.6	44.0	43.1	39.7	46.1	47.4	48.4	45.8	48.9	47.2	44.9	49.8
T_1 relaxation time	M	-	-	-	-	**1,936.5**	**1,940.0**	**1,940.0**	**1,929.2**	**1,944.0**	**1,941.7**	**1,944.0**	**1,945.0**	**1,946.9**	**1,943.7**	**1,950.0**	**1,945.7**
SUB right	SD					57.6	66.6	67.6	56.4	48.7	50.2	50.6	51.0	64.0	64.0	58.9	58.7
T_1 relaxation time	M	-	-	-	-	**2,091.7**	**2,092.5**	**2,097.4**	**2,098.5**	**2,112.5**	**2,109.1**	**2,109.1**	**2,109.5**	**2,109.4**	**2,108.3**	**2,110.9**	**2,106.5**
CA1 right	SD					56.5	61.8	51.3	47.2	37.6	40.5	37.4	38.3	37.5	38.1	40.5	33.9
T_1 relaxation time	M	-	-	-	-	**2,083.7**	**2,071.2**	**2,051.2**	**2,083.3**	**2,120.5**	**2,115.9**	**2,119.3**	**2,121.4**	**2,092.7**	**2,102.1**	**2,107.6**	**2,095.7**
CA2 right	SD					68.5	85.2	95.4	82.2	74.7	71.4	83.1	67.2	72.4	65.5	83.1	73.7
T_1 relaxation time	M	-	-	-	-	**1,929.2**	**1,942.5**	**1,925.0**	**1,930.6**	**1,959.1**	**1,956.8**	**1,962.5**	**1,963.1**	**1,950.0**	**1,946.9**	**1,962.0**	**1,945.7**
CA3 right	SD					74.7	63.9	70.7	70.0	68.4	78.4	74.7	72.3	56.6	48.0	54.3	41.7
T_1 relaxation time	M	-	-	-	-	**2,220.7**	**2,214.5**	**2,222.4**	**2,213.2**	**2,233.0**	**2,229.5**	**2,233.0**	**2,236.9**	**2,224.0**	**2,226.0**	**2,228.3**	**2,226.1**
DG/CA4 right	SD					40.3	41.1	42.4	42.5	50.2	52.1	53.1	54.6	42.0	40.7	37.9	40.2

Note. Values were calculated after exclusion of outliers using the outlier labeling rule (see Methods). Note that CTR only completed the cognitive assessment in order to control for test-retest effects induced by repeated testing. Cognitive component scores are not shown (see main article for an overview). BDNF is indicated in ng/ml, IGF-I and VEGF are shown in pg/ml. T_1 relaxation time is expressed in ms. BDNF = brain-derived neurotrophic factor, BIS = 'Berlin Intelligence Structure Test', CA = cornu ammonis, COMBO = group undergoing cycling exercise and maze training, CTR = passive controls, DG = dentate gyrus, DST = 'Digit Symbol Test', ERC = entorhinal cortex, ERGO = group undergoing cycling exercise, FRS = 'Fragebogen Räumliche Strategien' (questionnaire to assess spatial strategies), IGF-I = insulin-like growth factor-I, MAZE = group undergoing maze training, SUB = subiculum, VEGF = vascular endothelial growth factor.
